# Classification of Alzheimer disease using DenseNet-201 based on deep transfer learning technique

**DOI:** 10.1371/journal.pone.0304995

**Published:** 2024-09-06

**Authors:** Zia-ur-Rehman, Mohd Khalid Awang, Javed Rashid, Ghulam Ali, Muhammad Hamid, Samy F. Mahmoud, Dalia I. Saleh, Hafiz Ishfaq Ahmad

**Affiliations:** 1 Faculty of Informatics and Computing, Universiti Sultan Zainal Abidin (UniSZA), Terengganu, Malaysia; 2 Information Technology Services, University of Okara, Okara, Pakistan; 3 Department of CS and SE, International Islamic University, Islamabad, Pakistan; 4 MLC Lab, Meharban House, Okara, Pakistan; 5 Department of CS, University of Okara, Okara, Pakistan; 6 Department of Computer Science, Government College Women University, Sialkot, Pakistan; 7 Department of Biotechnology, College of Science, Taif University, Taif, Saudi Arabia; 8 Department of chemistry, College of Science, Taif University, Taif, Saudi Arabia; 9 Department of Animal Breeding and Genetics, Faculty of Veterinary and Animal Sciences, The Islamia University of Bahawalpur, Bahawalpur, Pakistan; University of Sargodha, PAKISTAN

## Abstract

Alzheimer’s disease (AD) is a brain illness that causes gradual memory loss. AD has no treatment and cannot be cured, so early detection is critical. Various AD diagnosis approaches are used in this regard, but Magnetic Resonance Imaging (MRI) provides the most helpful neuroimaging tool for detecting AD. In this paper, we employ a DenseNet-201 based transfer learning technique for diagnosing different Alzheimer’s stages as Non-Demented (ND), Moderate Demented (MOD), Mild Demented (MD), Very Mild Demented (VMD), and Severe Demented (SD). The suggested method for a dataset of MRI scans for Alzheimer’s disease is divided into five classes. Data augmentation methods were used to expand the size of the dataset and increase DenseNet-201’s accuracy. It was found that the proposed strategy provides a very high classification accuracy. This practical and reliable model delivers a success rate of 98.24%. The findings of the experiments demonstrate that the suggested deep learning approach is more accurate and performs well compared to existing techniques and state-of-the-art methods.

## Introduction

It is not just getting old and wrinkled that people worry about nowadays; dementia is also becoming a significant problem. The inability to recall familiar details and places due to dementia is quite concerning [1]. Dementia comes in a wide variety of forms: Alzheimer’s disease, vascular dementia, Lewy body dementia, frontotemporal dementia, Parkinson’s disease dementia, and mixed dementia [2]. AD is the most common kind of dementia [3]. AD is a degenerative brain disorder that destroys memory and other cognitive abilities over time, eventually leaving sufferers unable to recall even the most basic information. They are more unpredictable than other diseases in terms of their onset. At its most advanced, AD can diminish a person’s ability to remember and even cause death. It is one of the challenges faced by the healthcare sector in the twenty-first century [2].

**Fig 1:**
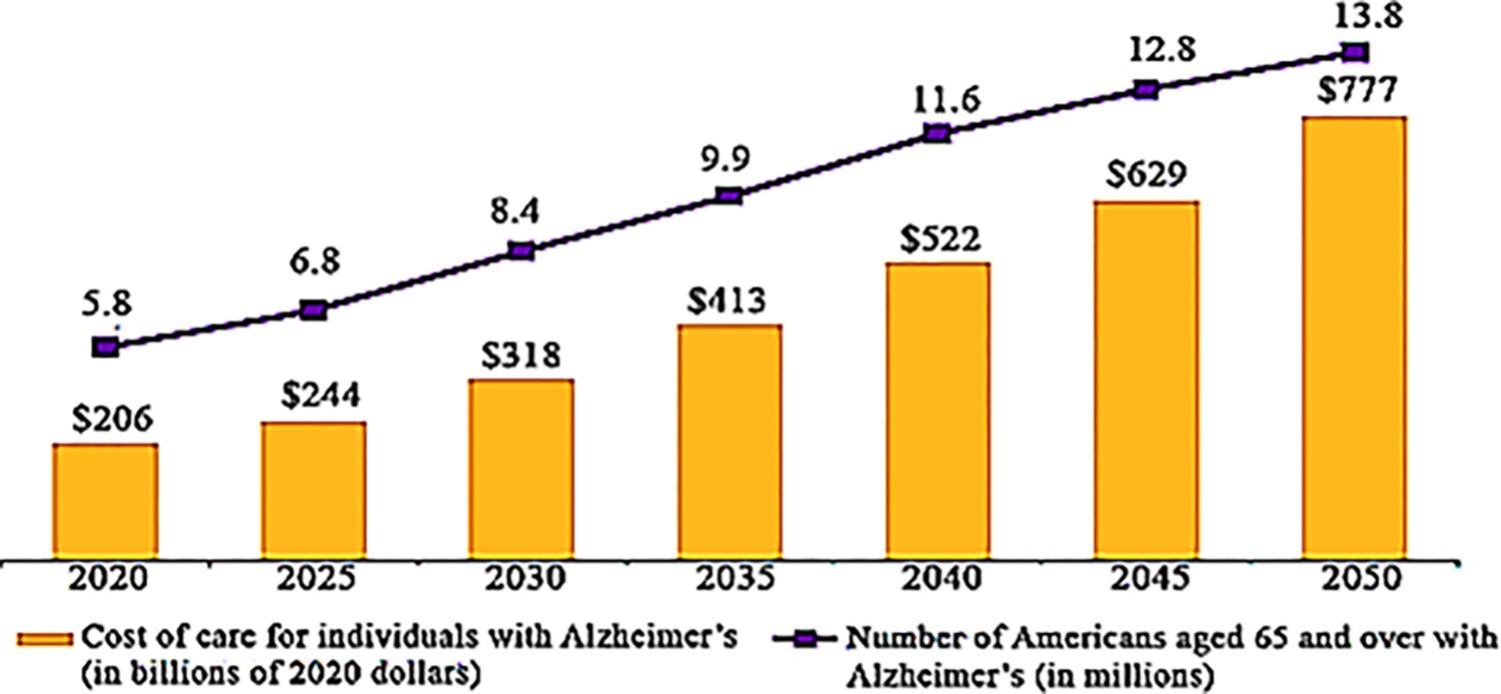
Cost and number of patients from 2020–2050 [10].

AD is currently the sixth leading cause of death in the United States, according to global figures [4]. Recent research shows this issue may rank third among senior citizens after cancer and cardiovascular illness [4]. By 2030, the WHO estimates that AD and other dementias will be responsible for 1.37 percent of all deaths globally [5]. According to the Alzheimer’s Society, there is currently no cure for AD as of 2019 [6,7]. Alzheimer’s affects approximately 5 million Americans; over 200,000 are under 65. According to the studies, by 2050, ten million adults over 60 will have AD [6,7]. According to 2019 research by AD Alzheimer’s Disease International (ADI), about 95% of the public is concerned they will develop AD in the future [8]. The financial burden of dementia should also be considered. The cost of AD in the UK was estimated at 26.3 billion in 2013 [9], with hospitalization costs accounting for 4.3 billion and diagnostic costs accounting for 85 million. Reducing the financial burden of AD is one of the primary benefits of early diagnosis. The cost and number of people diagnosed with Alzheimer’s from 2020–2050 are displayed in Fig 1.

Neuroimaging, assessments of behavior and mood, and cognitive tests are all used to identify early-stage AD. Dementia and brain pathology can be diagnosed with the help of neuroimaging techniques such as computed tomography (CT), positron emission tomography (PET), MRI, and single-photon emission computerized tomography (SPECT) [11]. Neuroimaging techniques such as MRI and CT are used to detect anomalies in the brain caused by disease [5,12]. There are not as many neurologists and neurosurgeons as there should be, so people with Alzheimer’s often have to wait weeks for an appointment. Especially in third-world nations like Pakistan, the doctor typically needs more diagnostic expertise. Therefore, it is essential to create a cost-effective and automatic diagnostic system for AD that would benefit patients and medical professionals.

Artificial intelligence (AI) now plays a vital role in modern life and healthcare. This domain’s cutting-edge field and method is deep learning (DL). Natural language processing (NLP), speech recognition [13,14], agriculture [15,16], facial recognition [17], smart cities [18], and healthcare [19] are just a few of the fields where DL has found use. Lung disease [20], medical imaging [21], mental disorder [22], breast cancer [23], skin lesions [24,25], and pancreatic tumors [26] are just a few of the medical conditions that benefit from the use of a DL.

DL is also used in AD, albeit with various datasets, methods, and class sizes. A multi-model convolutional neural network (CNN) was proposed by Liu et al. [27] to detect Alzheimer’s disease, mild cognitive impairment (MCI), and normal control (NC). To diagnose AD and its many stages, including MCI and full-blown dementia, Zhang et al. introduced a highly interconnected CNN equipped with a connection-wise attention approach trained on the ADNI dataset. The AD Neuroimaging Initiative (ADNI) dataset was mined for random images. The multi-model ensemble method attained an accuracy of 88.9 percent, outperforming competing models (DenseNet and multi-task deep convolutional neural network (DCNN)). Accuracy in classifying between cMCI and ncMCI was 787.82 percent, cMCI and NC were classified at 97.35 percent, and AD at 98.15 percent [28].

In order to detect AD, ghazal et al. [29] created a Transfer Learning (TL) method called AD detection with a transfer learning approach (ADDTLA) that is built on AlexNet and is trained on the Alzheimer’s dataset (4 classes of images). The authors correctly classified participants into one of four AD stages: MD, VMD, MOD, and ND, with 91.70 percent precision. Using the Alzheimer’s dataset, Ajagbe et al. [30] proposed deep CNN and TL models such as VGG16 and VGG19 (4 classes of images). Compared to VGG16 and CNN, VGG19 performed better in accuracy (77.66%) and AUC (81.55%).

The existing research has primarily focused on mild dementia (MD), very mild dementia (VMD), moderate dementia (MOD), and no dementia (ND) stages of AD. However, there is a significant lack of studies that specifically address the accurate diagnosis of severe dementia (SD) of AD. The literature needs a model trained on the AD5C dataset, which is a dataset collected from various hospitals in Lahore, Pakistan. This gap indicates a need for research efforts to utilize this dataset for AD diagnosis. The study aims to fill the gap in the literature by developing an automatic diagnosis system specifically for SD of Alzheimer’s. This focus on severe dementia contributes to a more comprehensive understanding and diagnosis of the disease. The researchers collected and established the AD5C dataset, which includes data from various hospitals. Using the AD5C dataset, the study leverages a diverse set of patient information, potentially leading to improved accuracy in AD diagnosis. The study introduces a deep transfer learning model named DenseNet-201. The proposed model outperforms the current state-of-the-art models in terms of accuracy. This performance improvement contributes to the advancement of Alzheimer’s diagnosis and can have significant implications for patient care and treatment decisions. In summary, the research addresses the lack of studies on SD of AD, utilizes the AD5C dataset, and improves the performance of AD classification models. These contributions enhance the accuracy and effectiveness of Alzheimer’s diagnosis, filling a research gap and offering potential benefits for patients and healthcare practitioners.

The following are the research contributions:

1. DenseNet-201 is applied as a deep transfer learning model to classify AD into the five established stages (MD, VMD, MOD, ND, SD).
2. Enhance the AD dataset with five classes to overcome the limited availability of the AD-labeled dataset by developing the AD5C dataset.
3. The performance of the proposed model increases in terms of accuracy.

A literature review is in Related Work, followed by the materials and methods, then results and discussion, and finally, the conclusion and future work.

## Related work

One of the most rampant types of neurological dementia is Alzheimer’s, which can lead to many brain disorders that affect memory. The early-stage AD diagnosis is complicated for doctors and researchers [31]. Numerous DL techniques and their architectures have been proposed to address various issues relevant to the brain. In the previous decades, researchers used different datasets, such as ADNI, open access series of imaging studies (OASIS), Australian imaging biomarker, lifestyle study of aging (AIBL), and Alzheimer’s dataset (4 class of images), etc., for AD classification and detection with a different number of classes and distinct methods.

Researchers [32] provided a multimodal AD diagnosis architecture (MADDi) to categorize Alzheimer’s in AD, NC, and MCI classes on the ADNI1 dataset. The model showed a 96.88% accuracy. Parmar et al. [33] stated a 3D CNN to differentiate Alzheimer’s in AD, Late MCI (LMCI), Early MCI (EMCI), and CN. This research collected data subjects from the ADNI database and obtained training at 99.4%, validation at 96.75%, and testing at 93% accuracy. Angkoso et al. [34] introduced a multiplane CNN (Mp-CNN) for Alzheimer’s classification in the AD, MCI, and NC classes on the ADNI-1 dataset subjects. The proposed model achieved 93.00% accuracy for multiclass AD-NC-MCI and better precision for NC, AD, and MCI at 95%, 93%, and 91%, respectively.

Hedayati et al. [35] proposed an ensemble of a pre-trained autoencoder and CNN; first, feature extraction was done, and then AD classification. The model classifies Alzheimer’s into AD, MCI, and NC, selected from the ADNI database. The proposed technique achieved accuracy for MCI/NC, AD/NC, and AD/MCI are 92.5%, 90%, and 95%, respectively. Saratxaga et al. [36] proposed 2DNet, 3DNet, and ResNet18 DL models on the subset of the OASIS-2 dataset, which classifies the AD into CN, VMD, MD, and MOD. The ResNet18 got the best balance accuracy (BAC), up to 93.18%, compared to other models. Jabason et al. [37] introduced an ensemble of hybrid DCNN and employed it on three classes, AD, MCI, and NC, on the OASIS-3 dataset. The attained accuracy was 95.23%. Hazarika et al. [38] used many DL models. The images were obtained from the ADNI (2020) dataset, classifying Alzheimer’s into CN, MCI, and AD. The DenseNet-121 got a better average accuracy, 90.22%, compared to other models.

Oktavian et al. [39] proposed a CNN technique with a residual network of 18 layers (ResNet-18). The images were acquired from the ADNI dataset and classified the disease into CN, AD, and MCI classes with 88.3% accuracy. Kannur et al. [40] Introduce a CNN model. The data was taken from the ADNI database, subjected to TL, and tested on five transfer learning techniques: VGG19, VGG16, Xception, InceptionV3, and NasNetMobile. The was tested on the CNN method and achieved a 91.17% accuracy in categorizing the AD, MCI, and CN. Abraham et al. [41] used a LeNet to classify the disease into AD, MCI, and NC stages. The dataset samples, magnetization prepared rapid gradient echo (MPRAGE) scans, were acquired from the ADNI database. The improved LeNet method got an average performance ratio of 96.64%.

The most common AD subtypes used in research are mild, moderate, and severe. There was not a single study we could find that accurately diagnosed AD in those who had SD. The need for a model that has been trained on the AD5C dataset is another problem with the current literature. According to the available literature, the current model’s performance may be superior concerning the third problem. With this research, we will get closer to solving these issues. Table 1 shows the summary of the related work.

**Table 1:** Summary of related work.

Ref.	Methodology	Alzheimer Classes	Dataset	Accuracy	Limitations
[32]	MADDi Model	AD, MCI, NC	ADNI-1	96.88%	• Imbalance dataset • Less stages of disease • Multimodal data
[33]	3D CNN Model	AD, LMCI, EMCI, CN	ADNI	93%	• fMRI Images • Only for 3D images
[34]	Multiplane CNN	AD, MCI, NC	ADN-1	93%	• Less stages of diseases
[35]	Ensemble of pre-trained AE and CNN	AD, MCI, NC	ADNI	95%	• Limited number of images. • Less stages of disease
[37]	Ensemble of hybrid DCNN	AD, MCI, NC	OASIS-3	95.23%	• Expensive with respect to computational cost • Less stages of diseases
[38]	DenseNet-121	AD, MCI, NC	ADNI-2020	90.22%	• Less accuracy • Less stages of disease
[39]	CNN with the Residual Network18 Layer (ResNet-18)	AD, MCI, NC	ADNI	88.3%	• Limited size of samples • Less accuracy
[40]	CNN Model	AD, MCI, NC	ADNI	91.17%	• Limited size of samples • Less stages of disease • Less accuracy • Used five pre-trained models.
[41]	An improved LeNet	AD, NC, MCI	ADNI	96.64%	• Limited size of samples • Less stages of diseases

## Materials and methods

Artificial Neural Networks (ANNs) are computational models that draw inspiration from the intricate network of neurons in the human brain. Machine learning and artificial intelligence (AI) benefit greatly from their ability to identify patterns in data, making them highly effective tools. An artificial neural network (ANN) is composed of layers of interconnected nodes or neurons, where each connection facilitates the transmission of signals between neurons. The recipient neuron undergoes signal processing and subsequently transmits messages to interconnected downstream neurons. The strength of artificial neural networks (ANNs) lies in their capacity to acquire knowledge and enhance performance through iterative learning processes. This learning process entails modifying the weights of the connections within the network by considering the discrepancy between the desired output and the output generated by the network [42]. The predominant learning process entails the utilisation of a technique known as backpropagation in conjunction with an optimisation algorithm, typically gradient descent, to minimise error or loss functions. Artificial neural networks (ANNs) can handle intricate patterns in extensive datasets, enabling their application in many tasks, such as speech recognition, image recognition, natural language processing, and more system [43]. Such networks can do DL.

**Fig 2:**
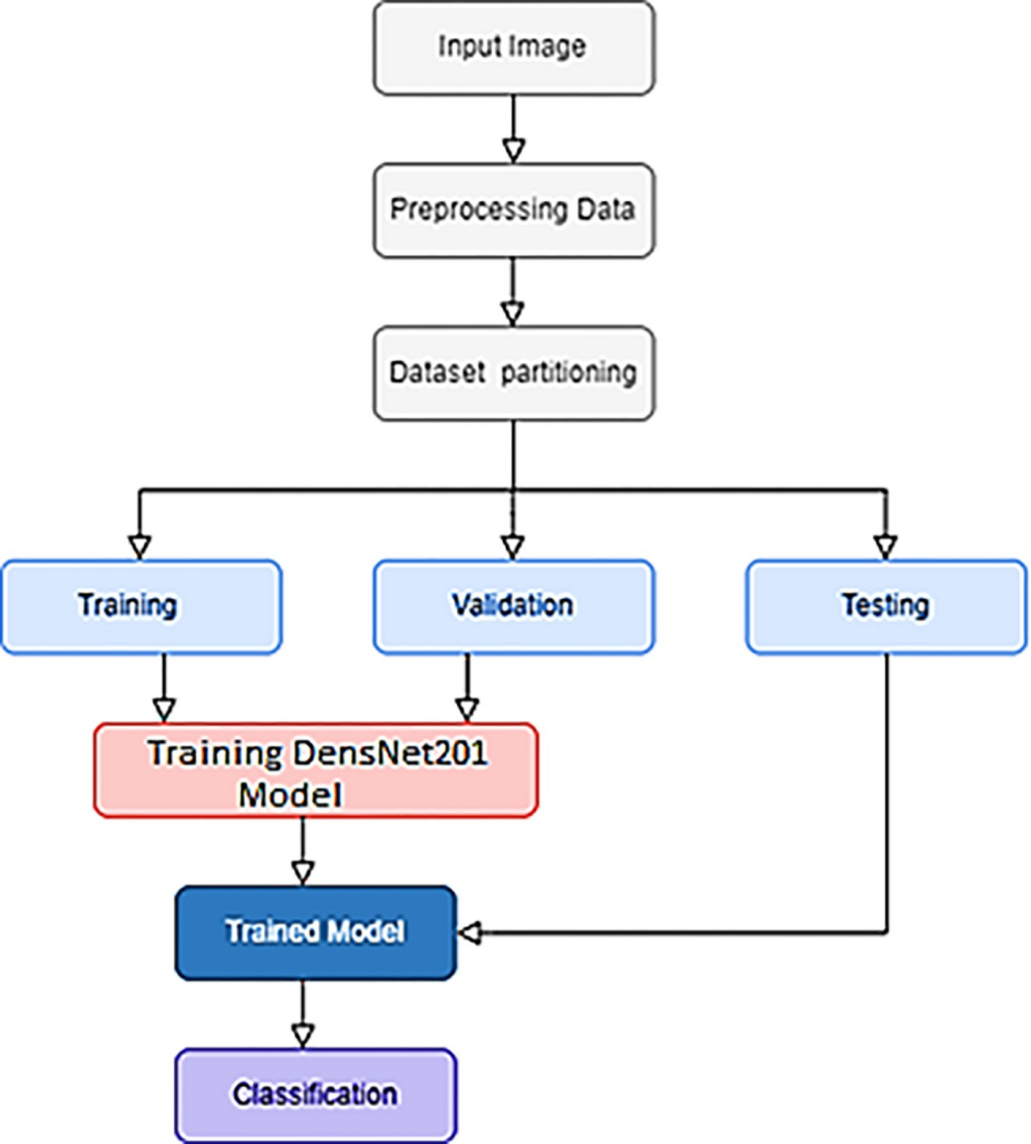
Flowchart of the proposed DenseNet-201 transfer learning model.

This research presents a DenseNet-201 approach based on the deep TL technique for classifying AD. Several pre-processing and augmentation techniques are employed initially to deal with the data set’s class imbalance problem. In the second stage, auto features are extracted, and a TL model (DenseNet-201) is implemented to classify AD. Fig 2 shows the flow chart for the proposed method.

### Dataset

DL techniques’ performance depends on the availability of an appropriate and reliable dataset. In this regard, the following dataset is used:

#### AD5C dataset

**Fig 3:**
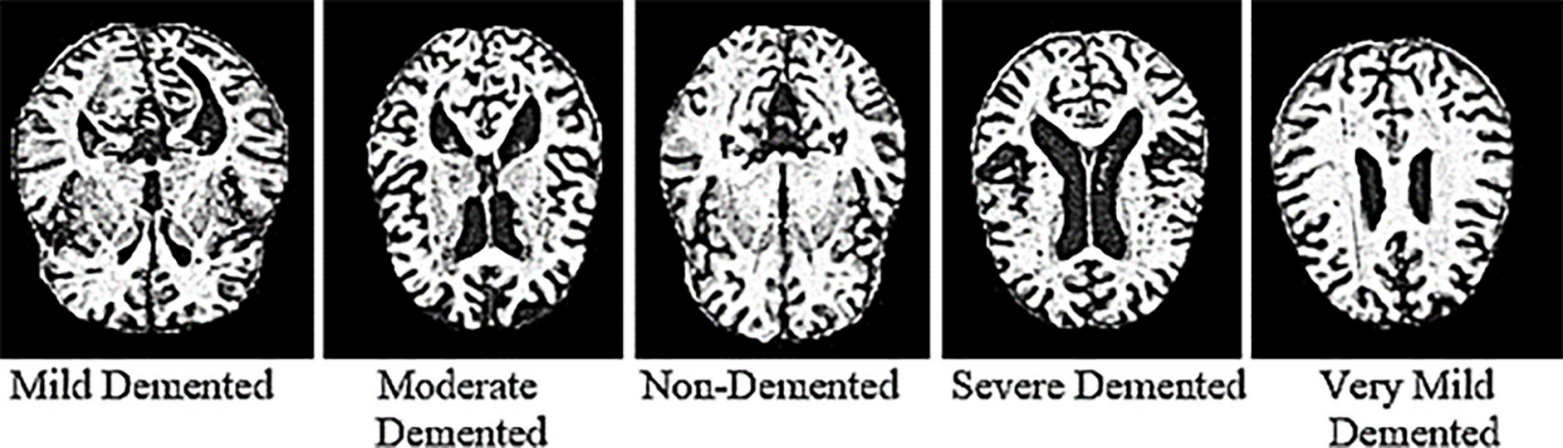
Different classes of AD5C disease dataset.

This work uses the” AD5C” dataset. The dataset has 2380 MRI images, including Alzheimer’s different stages scans as; 372 MD, 357 VMD, 637 MOD, 324 ND, and 691 SD. All dataset images are in jpg format; most images have 176 by 208 sizes, and some have varying sizes. The dataset was obtained from Kaggle [44]. Kaggle is an online organization for machine learning, deep learning, and data science practitioners. The information about the dataset is presented in Table 2. The images of the MD, MOD, ND, VMD, and SD classes were selected randomly from the whole dataset. Fig 3 shows the images of Alzheimer’s stages.

**Table 2:** Summary of AD5C dataset.

Class Names	No of Images
Mild demented (MD)	372
Very mild demented (VMD)	357
Moderate demented (MOD)	637
Non-demented (ND)	324
Severe demented (SD)	691
**Total Images**	**2381**

#### Data division

In this research, dataset was divided into three subsections: training, validation, and testing. As the term means, training parts refer to the data used during training time. In contrast, validation data is employed immediately following a training epoch’s output to determine the training’s effectiveness. The testing part comprises an untested set of data applied to check a classifier’s reliability after completing training and validation.

The dataset was spliced into 80% training, 10% testing, and 10% validation data. The AD5C dataset was divided into 1904, 239, and 239 training, validation, and testing images, respectively, as shown in Table 3. The training set consisted of 298 images for MD, 285 MOD, 509 VMD, 258 ND, and 553 for SD class. The validation and testing set contained 37 MD, 36 MOD, 64 VMD and 33 for ND and 69 for SD classes.

**Table 3:** AD5C dataset splitting.

Split	Classes	No of Samples	Total Samples
Training	MD	298	1903
	MOD	509	
	VMD	285	
	ND	258	
	SD	553	
Validation	MD	37	239
	MOD	64	
	VMD	36	
	ND	33	
	SD	69	
Testing	MD	37	239
	MOD	64	
	VMD	36	
	ND	33	
	SD	69	
**Total**			**2381**

### Pre-processing

Pre-processing is used for all of the input images of the AD5C dataset to get classification results that are more reliable and have better features. Pre-processing methods are typically employed to prevent image degradation due to the increasing pixel intensity and contrast. This research suggested pre-possessing the data used to train and test the suggested DenseNet-201 model. The CNN method needs a significant amount of recurrent training; a large image dataset was needed to avoid the over-fitting problem; for this purpose, different augmentation techniques are used in this research.

#### Image resizing

In this work, Image resizing is done for the uniformity of the dataset. The AD5C dataset generally comprises images with dimensions of 176 × 208, although there are a few instances where the dimensions are 182 × 208. The dimensions of the dataset have been changed to 224 × 224 with Python code.

#### Data augmentation

Scale transformation, rotation transformation, shear transformation, width shift range transformation, height shift range parameter, random zoom transformation, flip parameter, brightness transformation, and channel shift transformation, techniques have been used on the training set to reduce over-fitting and enhance the dataset’s diversity. A disproportional dataset balanced with an augmentation procedure produces favourable results. It simplifies early Alzheimer’s diagnosis. In this study, the dataset size was artificially increased using the image data generator method of the Keras library in Python. Scale transformation reduced computing time by applying minor pixel values within a similar range. With the parameter value (1./255), every pixel has a value ranging from 0 to 1. The rotation transformation was also utilized to change the angle of the images, so twenty-five degrees were employed to rotate the angular position.

Shear transformation involves fixing one image axis and shifting the other to a shear degree, such as 0.2 in this scenario. The width shift range transformation, adjusted to 0.1 value, could move images freely to the left or right. At the same time, the height shift range parameter adjusted training images perpendicularly at 0.1 value. A random zoom transformation was applied using the zoom range input, where a value larger than 1.0 indicates zoomed in, and a below 1.0 indicates de-magnification of the images. Thus, the image was enlarged using a 0.2 zoom range. The image was horizontally flipped using the flip parameter. The brightness transformation was applied, with 0.0 indicating zero brightness and 1.0 denoting the highest brightness, resulting in a zoom range of 0.5 to 1.0. The channel shift transformation randomizes channel values within a range. The fill mode was closest to the 0.05 channel shift range.

### DenseNet-201 architecture

Convolutional Neural Networks (CNNs) have established a strong reputation in the field of image data processing, producing superior results compared to traditional methods. However, they require enormous data for the training phase, sometimes socially referred to as data-hungry algorithms especially training from scratch. However, transfer learning (TL) has solved this problem to a very large extent, where a pre-trained model is retrained to perform a specific task with fewer data samples. Pre-trained deep learning models provide substantial benefits in artificial intelligence and machine learning. These models save practitioners time and computing resources by providing powerful starting points for numerous tasks, leveraging knowledge from prolonged training on big and diverse datasets. The ability to adapt pre-trained models to specific tasks using limited labelled data is a major feature of transfer learning. It reduces the requirement for enormous datasets. Pre-trained models are flexible because they can parse many kinds of data for useful hierarchical characteristics. Additionally, these models are useful for practitioners in several areas due to their resistance to overfitting and rapid convergence during fine-tuning. Efficient deployment of complex architectures in various applications, from computer vision to natural language processing, is made possible by the availability of pre-trained models from well-established repositories, encouraging community cooperation. In general, pre-trained deep learning models have the potential to democratize access to cutting-edge machine learning technology, be more efficient, and have high transferability.

The selection of the best TL model is crucial, as different models may perform better or worse depending on the dataset. One approach to address this enigma is employing top networks to achieve good results. In this research, we employed six widely recognized pre-trained models, ResNet34, ResNet50, VGG16, VGG19, AlexNet, and MobileNetv2—to evaluate their performance in classifying different stages of Alzheimer’s disease. To this end, DenseNet-201 has been used in this research. The layers of a dense convolutional network (DenseNet) are all connected in a feed-forward manner. Typical CNNs, on the other hand, have L connections between their layers, where each pairwise connection is proportional to L (L + 1)/2. Regarding the number of filters per layer, DenseNet only uses 12, and its number of feature maps is somewhat limited [43]. DenseNet has benefits in simplifying gradients, feature repetition, parameter reduction, and feature deployment [45].

**Fig 4:**
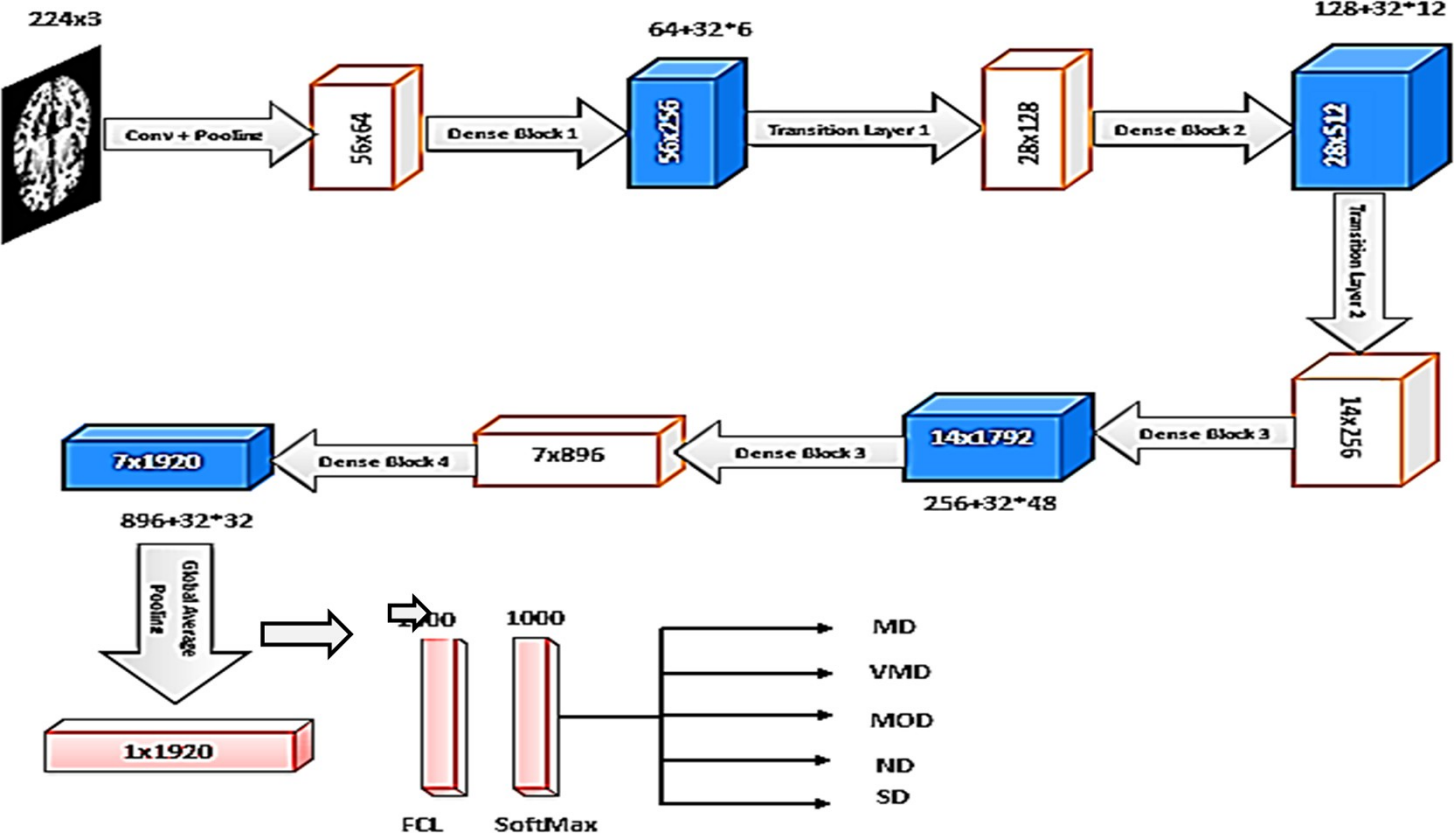
Transfer learning based DenseNet-201 building block.

The TL model is constructed using the keras library. The DenseNet-201 is a 201-layer CNN. It uses the AD5C dataset. The network will classify images into five stages of AD, including MD, VMD, MOD, ND, and SD. The input images are resized to 224×224 pixels, and the model is trained with a batch size of 32. We start with the DenseNet-201 architecture as the base model. By setting include top to false, we exclude the top classification layer, which allows us to add our layers for fine-tuning. Every layer includes rectified linear unit (ReLu) activation convolution with a 3×3 filter and batch normalization (BN), as shown in Fig 4.

For each block, an image pixel is used as an input in the type of a matrix. This matrix then passes to the batch normalization phase, which helps to decrease the over-fitting problem while the model is being trained. ReLu activation function to convert x to 0 if it is negative, but not if it is not smaller than zero. Convolutional with a 3×3 filter will be multiplication by a convolution matrix size with a 3x3 filter to generate a matrix image crossing the ReLu activation step. The outcome is a previously processed matrix value. A global average pooling layer is applied to the output of the base model. This pooling operation reduces the spatial dimensions of the feature maps while preserving the most important information. A fully connected dense layer with 128 units and a ReLU activation function is added to the global average pooling layer. This layer helps to extract higher-level features and introduces non-linearity into the model. The final dense layer is added with n class (5 in this case) units, representing the number of classes in the classification task. The SoftMax activation function is used to obtain the predicted class probabilities. The model is compiled with a categorical cross-entropy loss function, Adam optimizer, and accuracy metric. This configuration allows the model to optimize its parameters based on the provided training data and evaluate its performance during training.

The major reason for selecting the DenseNet-201 for AD classification is that it has shown great promise in accurately classifying AD. Whereas other techniques rely on larger and more complex techniques, DenseNet-201 is a simple network that achieves high accuracy with fewer parameters [46]. One of the key advantages of DenseNet-201 is its ability to learn and extract more features than other deep learning models, such as ResNet18, VGG16, Inception-v3, and Xception, which help to improve the accuracy of AD classification [47,48]. DenseNet-201 has demonstrated superior performance in AD classification with lesser computation cost. Using DenseNet-201 for AD classification offers a significant advantage over other methods, providing higher accuracy, less complexity, and resource utilization. The parameters used in the DenseNet-201 model experiment are shown in Table 4.

**Table 4:** Configuration parameters used in the experiment.

Parameters	Values
Architecture utilized	DenseNet-201
Type of transfer	From scratch transfer Knowledge
Train Layers	All
Learning Algorithm	Adam
Learning Rate	0.0001
Activation Function	ReLu & SoftMax
Loss Function	Categorical-Cross-entropy
Batch Size	32
Epochs	50
Number of Classes	5 (MD, VMD, MOD, ND, and SD)

### Assessment measures

The accuracy, F1-score, recall, and precision were used to assess the model’s effectiveness. Following is a detailed of the performance measures used in this study.

#### Accuracy

The proposed model accuracy is assessed by the number of accurate predictions as a percentage of all correctly predicted.

$$Accuracy=\frac{TP+TN}{\left(TP+TN+FP+FN\right)}$$ (1)

Where TP = True Positive, TN = True Negative, FP = False Positive, FN = False

#### Precision

Precision means that we can find inconsistencies when using the same tool over and over, like when evaluating the same part. One such metric is precision, defined as the proposed model achieving precision by dividing the amount of true positive instances by the total amount of positive predictions.


$$Precision=\frac{TP}{TP+FP}$$ (2)

*Recall*. Another essential metric is recall, described as splitting input data into classes that the algorithm accurately predicts.


$$Recall=\frac{TP}{\left(TP+FN\right)}$$ (3)

#### F1-score

The F1-score is a widely used measure that combines recall and precision into a single value. The F1-score is computed as follows:

$$F1-score=\frac{2*(Precision*Recall)}{\left(Precision+Recall\right)}$$ (4)

#### ROC curve and AUC score

The receiver operating characteristic (ROC) is a likelihood curve, and the area under curves (AUC) shows the extent of distinction. The cutoff threshold for an excellent model can be found with the help of the widely used ROC curve.

## Results and discussion

Google Colab Pro [49] account was used to experiment with the DensNet201 model. The DenseNet-201 method was applied in Python with open-source Keras [50] libraries. In the training phase, the optimizer for the given model, Adam, and a learning rate 0.0001 were used. Because it was a multiclass classification task, categorical-cross-entropy was chosen as the loss function. The batch size was 32, and the epoch frequency was 50. The model was trained from scratch. The experimental results concentrated on the following:

- The performance of the proposed DensNet201 model was evaluated without a data augmentation technique.
- The performance of the proposed DensNet201 model was evaluated with a data augmentation technique.
- The results of the proposed method are compared with the existing studies.
- The state-of-the-art approaches were compared to the proposed DensNet201 model results.

### Performance of the proposed DenseNet-201 on AD5C dataset without applying data augmentation techniques applied on training set

In this research, we conducted two experiments. In the first experiment, data augmentation techniques were applied to the training set, and in the other experiment, data augmentation techniques were not applied to the training set.

**Fig 5:**
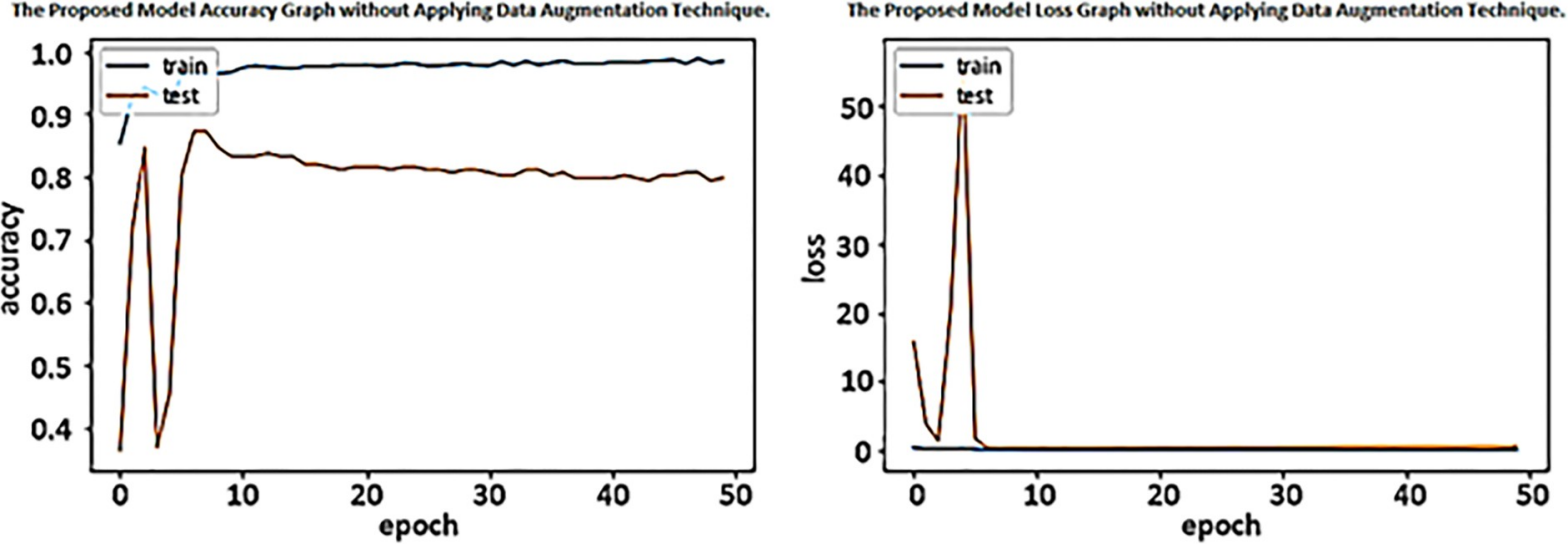
Accuracy and loss graph of the proposed model without data augmentation techniques.

The first experiment evaluated the performance of the presented DenseNet-201 framework without performing data augmentation techniques on the training set of the AD5C dataset. The accuracy and losses in each epoch in training and testing are depicted in Fig 5. Fig 5(A) shows that the training accuracy of the presented method increases quickly at the start and increasingly reaches 98% to 50 epochs. The validation accuracy increases rapidly until the eighth epoch and reaches 88%, then decreases and obtains less than 80% after 50 epochs. Fig 5(B) demonstrates less than one percent training loss from epoch 1 to epoch 50; however, validation loss increases by 50% after epoch three and becomes stable after epoch 7. The proposed technique achieved 96.92% accuracy on test data (unseen data), as shown in Table 5.

**Table 5:** Classification accuracies, recall, precision and F1-score of the proposed model applying data augmentation techniques on AD5C dataset.

Perf. Measures	Mild Demented	Moderate Demented	None Demented	Severe Demented	Very Mild Demented	Average Accuracy
Accuracy	90.63%	95.31%	96.88%	100%	100%	96.92%
Precision	97%	98%	94%	96%	100%	-
Recall	91%	95%	97%	100%	100%	-
F1-score	94%	97%	95%	98%	100%	-

Table 5 shows the results of applying the proposed DenseNet-201 model to the AD5C dataset without using data augmentation approaches regarding classification accuracy, precision, recall, and F1-score. The AD5C dataset is a collection of MRI and CT scan pictures of the brain that were collected to research AD. The results are divided by dementia severity: VMD, MD, MOD, ND, and SD. The chart also offers an overall measure of the model’s performance across all classes, the average accuracy metric. The proportion of successfully identified samples for each category may be seen in the accuracy column. The proposed model, for instance, can get an accuracy of 90.63% for moderately demented samples, 95.31% for mildly demented samples, 96.88% for non-demented samples, 100% for severely demented samples, and 100% for very mildly demented samples. Across all categories, the model achieves an average accuracy of 96.92%. The precision column shows the percentage of positive “positive” samples. A smaller percentage of false positives corresponds to a higher precision rating. Except for the Very Mild Demented category, the precision values for all other categories are listed below. The recall metric shows the percentage of positive samples that were properly anticipated out of all observed positive samples. A lower rate of false negatives corresponds to a higher recall value. Except for the Very Mild Demented group, recall values are given for all other groups. The F1-score column is a combined statistic of precision and recall that provides a more comprehensive assessment of the model’s efficacy. Accuracy is measured by taking the harmonic mean of accuracy and recall. Except for the VMD group, the F1-scores are supplied. The accuracy, precision, recall, and F1-score for each class of dementia severity are displayed in Table 5 for the proposed DenseNet-201 model’s performance on the AD5C dataset. These results show that the model performs well, especially when correctly identifying samples from different dementia severity categories, which could help in the future diagnosis and understanding of AD.

**Fig 6:**
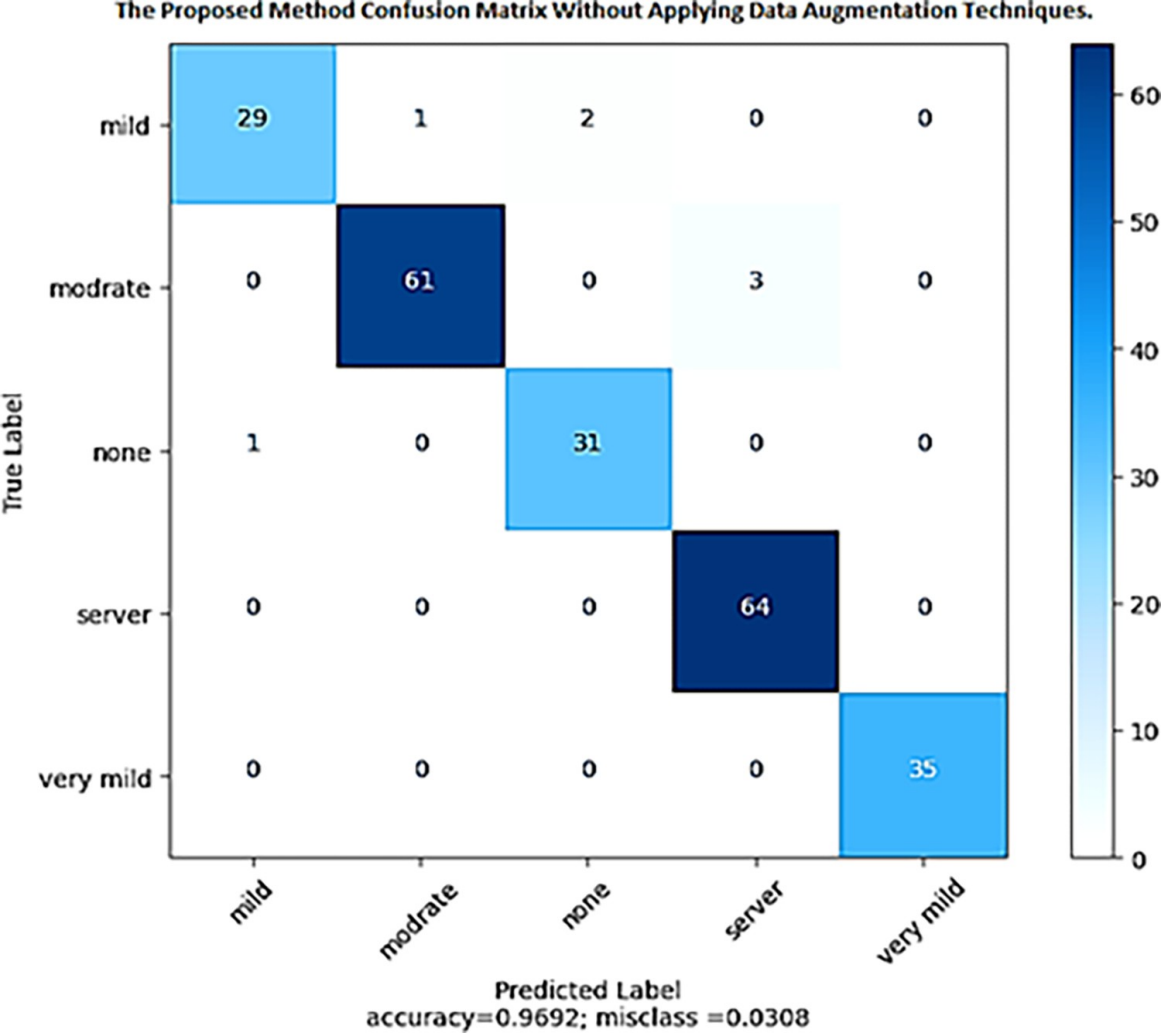
Confusion matrix of the proposed method without data augmentation techniques.

We can see the proposed DenseNet-201 model’s confusion matrix on the test set (without any data augmentation approaches) in Fig 6. The confusion matrix shows how well the model’s predictions match the true labels for each of the five levels of dementia severity (mild, moderate, none, severe, and very mild). The confusion matrix displays the predicted class labels with the actual class labels in rows and columns, respectively. The numbers in the matrix represent the total number of samples that meet the criteria for each possible set of true and false predictions. For instance, the Mild Demented class had 29 correctly categorized samples in the first row of the confusion matrix. None of the samples were misclassified as severely demented or mildly demented, but one was wrongly labeled as moderately demented, and two as none demented.

In the same way, all 61 samples from the moderately demented class fell into that category in the second row. The severity of dementia was incorrectly assigned to 3 samples. The model’s overall performance across all classes can be seen in the confusion matrix. It draws attention to how often each class is correctly predicted and how often it is incorrectly classified. The confusion matrix can evaluate the model’s performance in correctly categorizing varying degrees of dementia. The model is said to have achieved an overall accuracy of 96.92% on the validation set. This metric represents the proportion of the test set that was correctly labeled. According to the data, the misclassification rate is 3.08% (100%—accuracy). Incorrectly classified test set samples are represented by this value. Fig 6 shows the confusion matrix, a useful tool for analyzing the model’s performance and identifying areas for improvement.

**Fig 7:**
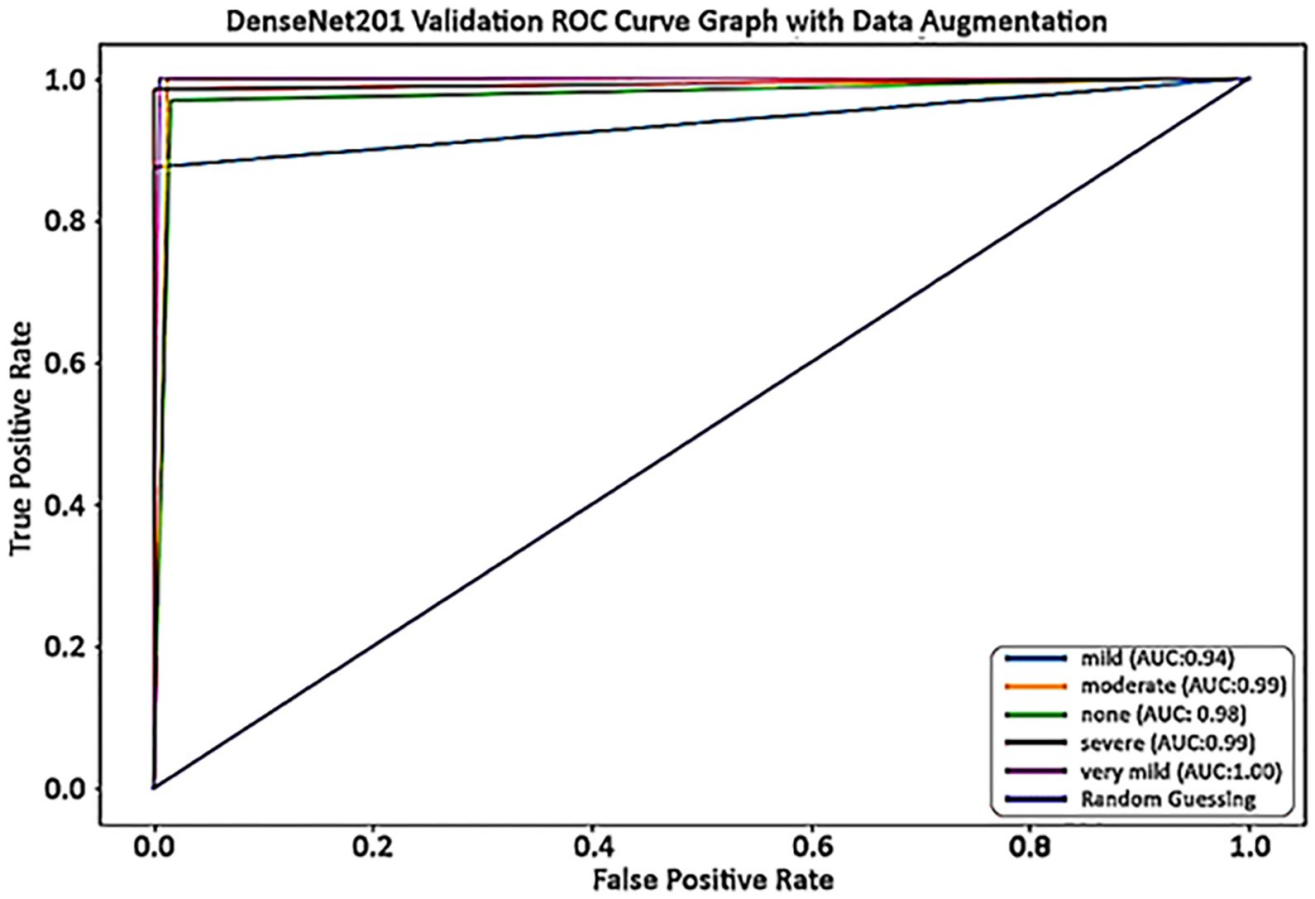
The proposed method ROC curve without applying data augmentation on AD5C dataset.

When the threshold for discrimination in a classification process is changed, the system’s diagnostic efficiency is depicted graphically as a receiver operating characteristic curve. In short, it is called the ROC curve. Fig 7 depicts the ROC curve that evaluates the system’s performance without applying data augmentation techniques. The ROC curve is used to demonstrate the specificity-sensitivity tradeoff. The proposed model’s ROC curve indicates how well the model distinguishes among MD, MOD, ND, SD, and VMD classes. Classifiers with top-left curves perform well, and random guessing produces diagonal points.

In the proposed model ROC, blue represents the random guessing, light blue depicts mild with 94%, orange color shows moderate with 99%, green color describes none with 98%, red depicts severe with 99%, and grey shows very mild with 100% accuracy. The ROC curve shows that the DenseNet-201 model executes as predicted. Moreover, the ROC curve exhibited that whole classes performed exceptionally well. The DenseNet-201 method showed better classification results in validation and test set classes with a higher AUC of 97.36%. The performance measures, such as F1-score, recall, accuracy, and precision, showed that the suggested technique was remarkably excellent on the AD5C dataset once the data augmentation approaches were applied to the training set.

The proposed DenseNet-201 model achieved promising results without applying data augmentation techniques on the AD5C dataset, but the proposed model’s training showed an overfitting problem during training. We can overcome the problem of overfitting by enhancing the dataset using different data augmentation techniques applied to the training set.

### Performance of the proposed DenseNet-201 on AD5C dataset applying data augmentation techniques applied on training set

**Fig 8:**
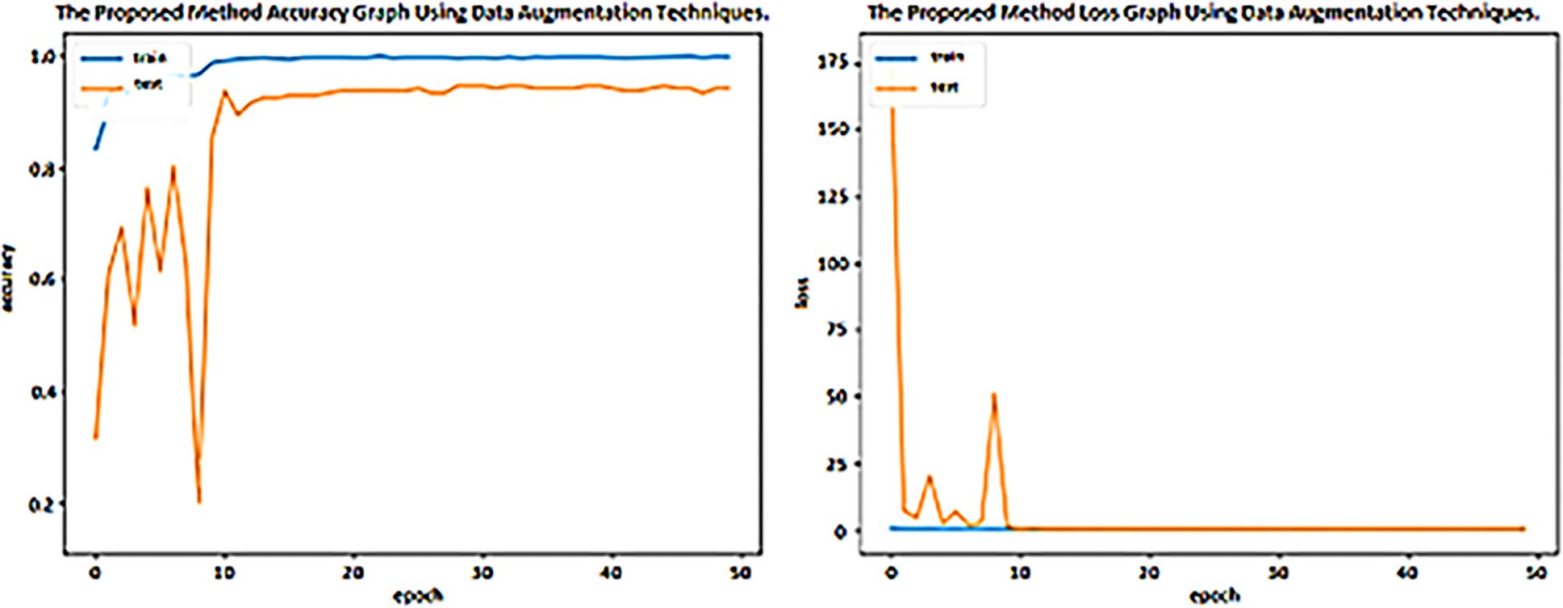
Accuracy and Loss graph of DenseNet-201 model applying data augmentation techniques.

The performance of the proposed model is evaluated by applying the data augmentation techniques applied on the training set of the AD5C dataset. The accuracy and losses in each epoch in training and testing are depicted in Fig 8. Fig 8(A) shows that the training accuracy of the presented method increases quickly at the start and increasingly reaches 99% to 50 epochs. The testing accuracy increases rapidly until ten epochs, reaching 97% after 50 epochs. Fig 8(B) demonstrates less than one percent training loss from epoch 1 to epoch 50; however, validation loss increases by 50% after epoch three and becomes stable after epoch 10. The proposed technique achieved 98.24% accuracy, as shown in Table 6.

**Table 6:** Classification accuracies, recall, precision and F1-score of the proposed model applying data augmentation techniques on AD5C dataset.

Perf. Measures	Mild Demented	Moderate Demented	None Demented	Severe Demented	Very Mild Demented	Average Accuracy
Accuracy	87.5%	100%	100%	100%	100%	98.24%
Precision	100%	98%	91%	100%	100%	-
Recall	88%	100%	100%	100%	100%	-
F1-score	93%	99%	96%	100%	100%	-

In Table 6, we can see the results of applying the proposed DenseNet-201 model to the AD5C dataset with the help of data augmentation approaches in classification accuracy, precision, recall, and F1-score. The suggested model has a 100% success rate with moderately demented samples and an 87.5% success rate with mildly demented samples. Across all categories, the model has an average accuracy of 98.24%. The precision column shows the percentage of true positives among all positive predictions. Values closer to one another on the accuracy scale suggest fewer false positives. The precise values for all classes except very mild demented are listed above. The recall column shows the percentage of true positives that were accurately predicted. The rate of false negatives decreases as recollection increases.

Each category, except for “VMD,” has its recall value listed. The F1-score column comprehensively evaluates the model’s efficacy by combining the two separate precision and recall metrics. It is determined by averaging the recall and accuracy of a measurement. F1-scores are supplied for all other categories except for the VMD category. The following table displays the enhanced performance of the DenseNet-201 model after data augmentation techniques were applied. The model’s strong accuracy, precision, recall, and F1-scores across the board for all categories of dementia severity attest to its efficacy in this regard. Overall, the model performs very well, as evidenced by its average accuracy of 98.24%.

Data augmentation improves the model’s robustness to fluctuations and generalizability to new data, as seen in Table 6. Increased confidence in the proposed DenseNet-201 model’s ability to correctly categorize the severities of dementia in the AD5C dataset is provided by this enhancement. The performance measures also demonstrate that the proposed model can classify Alzheimer’s efficiently and that the system’s overall performance is good.

**Fig 9:**
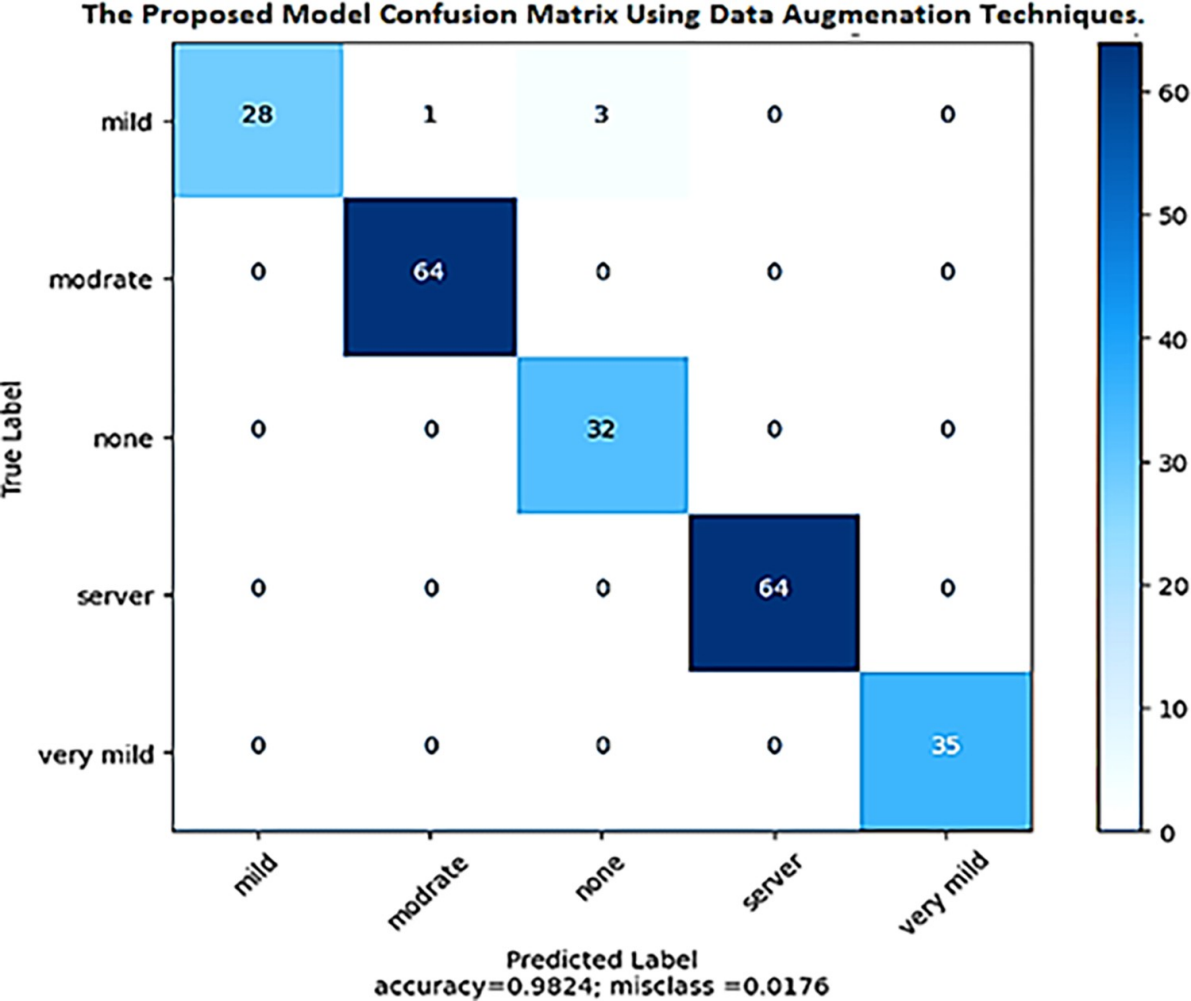
Confusion matrix of the proposed method applying data augmentation techniques.

As shown in Fig 9, the suggested DenseNet-201 model generates a confusion matrix of the test set when applying data augmentation methods to the training set. There were 28 examples from the MD class that could be accurately placed in the first row of the confusion matrix. One sample was incorrectly categorized as MD, and three others as ND. No samples were incorrectly labeled as either very mild or SD. Similarly, all 64 samples from the MOD class in the second row were accurately categorized as MOD. On average, the model was found to be 98.24% accurate across the validation set. Correctly identified samples represent this statistic as a fraction of all test set samples. The misclassification rate, calculated as 100% minus accuracy, is claimed to be 1.76%. This figure indicates the fraction of test samples that were misclassified. Fig 9 shows the confusion matrix, which may be used to understand how well the suggested DenseNet-201 model performs with data augmentation. It exemplifies the model’s high overall accuracy and low misclassification rate in predicting outcomes across many dementia severity classifications. These results show the model’s accuracy in identifying dementia severity and highlight its potential clinical utility in diagnosing AD.

**Fig 10:**
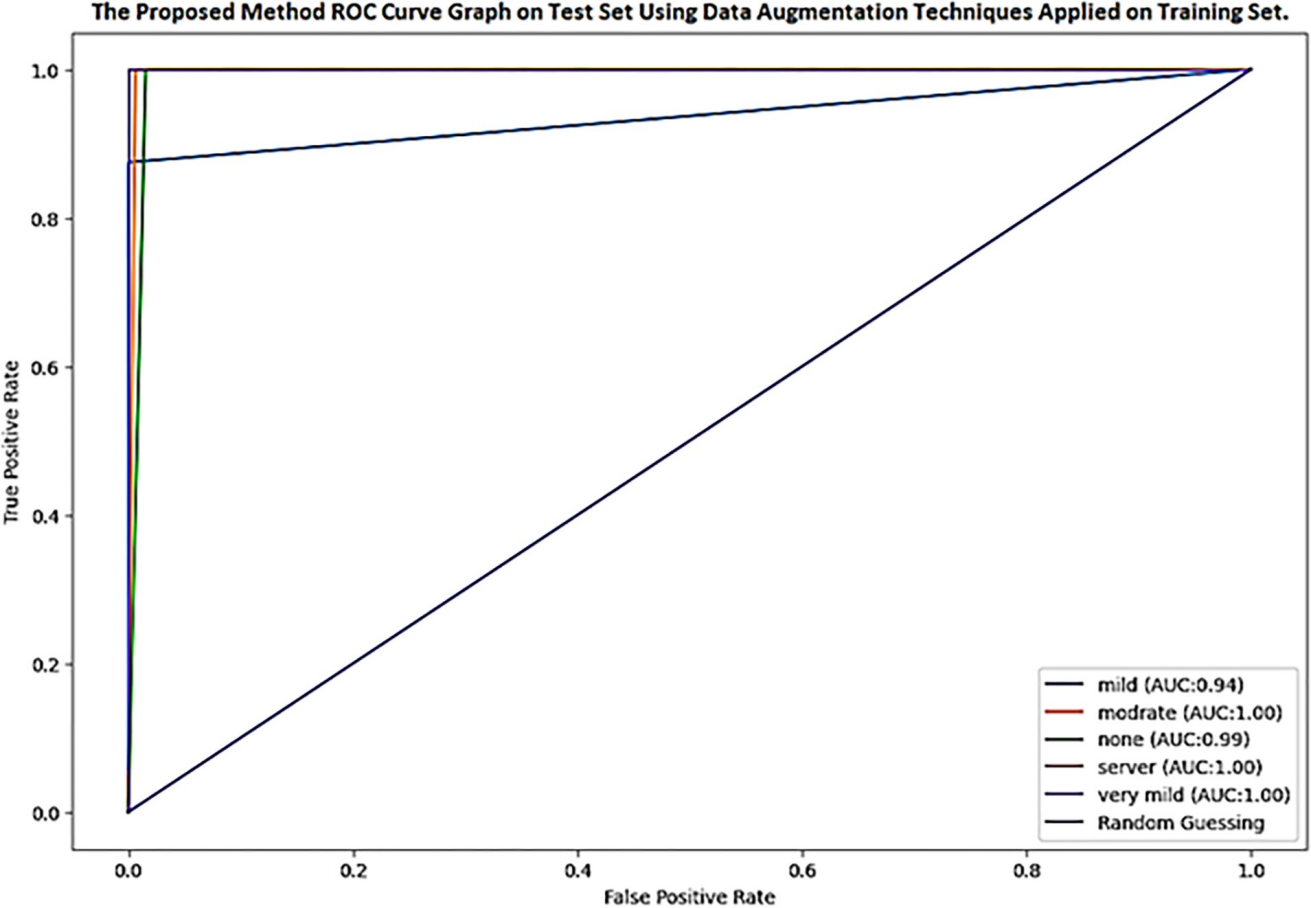
The proposed method ROC curve on test set applying data augmentation techniques.

Fig 10 depicts the ROC curve on the test set used to evaluate the system’s performance when data augmentation techniques were used. In the proposed model ROC, blue represents the random guessing, light blue depicts mild with 94%, orange color shows moderate with 100%, green color describes none with 99%, red depicts severe with 100%, and grey shows very mild with 100% accuracy. The ROC curve shows that the DenseNet-201 model executes as predicted. Moreover, the ROC curve exhibited that whole classes performed exceptionally well. The DenseNet-201 method showed better classification results in validation and test set classes with a higher AUC of 98.24%.

The performance measures, such as F1-score, recall, accuracy, and precision, showed that the suggested technique was remarkably excellent on the AD5C dataset once the data augmentation approaches were applied to the training set.

The following recommendations were suggested for improving the proposed method’s performance. The quantity of images in each class varies over the sample, demonstrating class imbalance. In order to deal with this problem, it is suggested to use data augmentation techniques to increase the number of samples in the underrepresented classes (MD, VMD, ND, and SD). As a result, the model’s efficiency and capacity to generalize to under-represented groups may be enhanced. Using cross-validation methods, such as k-fold cross-validation, to get a more accurate estimate of the model’s performance is recommended for ensuring the results’ robustness. It is useful for testing the model’s capacity to extrapolate results. The performance of the proposed method can be improved by addressing the recommendations mentioned above.

Using transfer learning, we first determined the various stages of AD. Here, we used DensNet-201 for transfer learning to classify AD into its stages: mild, moderate, advanced, and severe. The second goal was to improve the AD dataset; earlier research had only used datasets with a maximum of four classes. We used the AD5C dataset, which includes five stages of AD (MD, VMD, MOD, ND, and an extra stage SD), for this purpose. Finally, we aimed to improve the proposed model’s performance accuracy. Compared to the state-of-the-art model, the suggested transfer learning model achieved the greatest accuracy of 98.24% in completing this job.

### Comparison with existing techniques

To compare the performance of the proposed method, we compared the results of the proposed model to existing approaches. In this study, a dataset AD5C was utilized, which had not previously been used in any research. So, no comparison is possible because this dataset has five classes, and others do not have SD classes. However, here we are comparing the proposed method with other state-of-the-art methods, which used a different number of classes, such as two, three, and four, and different datasets. It was found that the suggested DL architecture outperforms the existing methods. Classifying AD using deep learning, Table 7 compares several approaches. Maqsood et al. [51], using the OASIS dataset, researchers classified AD into stages using a pre-trained CNN named AlexNet. The approach obtained 74.27% for precision, 74.27% for recall,82.53% for F1-score, and 92.85% for accuracy. Multiple datasets (ADNI, OASIS, AIBL) were used for the TL and inception of ResNet-V2 implementation for the AD and NC classes in [52]. The method had a 95% success rate on the AIBL dataset, with a sensitivity of 88.1% and a specificity of 96.4%. Using the AD neuroimaging initiative (ADNI) and the AD, brain imaging, and biomarkers library (AIBL) datasets, the DA-MIDL technique was first described in [53]. The method attained 92.4% accuracy, 91.0% sensitivity, 93.8% specificity, and 96.5% area under the curve. Ajagbe et al. [30] used VGG16 and VGG19 models on a four-class Alzheimer’s dataset (MD, VMD, MOD, ND) VGG19 achieved 77.66% accuracy, 81.55% AUC, 45.05% F1-score, 58.48% precision, and 36.67% recall. Using the same Alzheimer’s dataset, Pradhan et al. [54] used the VGG19 and DenseNet169 models. Using VGG19, the method attained an accuracy of 82.6% and an AUC of 86.7% for Alzheimer’s dataset classification of MD, MOD, ND, and VMD. Ghazal et al. [55] proposed ADDTLA, a CNN-based technique. The approach got 96% accuracy, 96.57% sensitivity, and 98.29% specificity. In order to distinguish between those with MCI and those with CN using the ADNI dataset, [56] presented CADx. Accuracy of 92% and F1-scores of 90% were all attained using this methodology. Last, the AD5C dataset was used by the suggested approach in the comparison table to categorize MD, VMD, MOD, ND, and SD. It got an F1-score of 98.2%, a precision of 97.8%, and a recall of 97.6%. The proposed DensNet121 model acquired 98.24% accuracy on the AD5C dataset. We found that the proposed DenseNet121 model had the highest accuracy (98.24%) and that VGG16 had the lowest (77.66%) on the test set of the AD5C dataset, compared to all the tested existing models. According to the previous research results, after evaluation of different metrics and comparison with other models, our DenseNet-201 model had superior accuracy, as shown in Table 7.

**Table 7:** Comparison with existing methods.

Ref., Year	Methodology	Classes	Dataset	Performance measures
[51], 2019	Pre-trained CNN, Alex Net	ND, VMD, MD, and MOD	Images from OASIS	Accuracy: 92.85%, Precision: 74.27%, Recall: 74.27%, F1-score: 82.53%
[52], 2022	Inception ResNet-V2 and TL	AD, NC	ADNI, OASIS, MIRIAD and AIBL	Accuracy: 94.20%, Sensitivity: 88.1%, Specificity: 96.4% on AIBL.
[53], 2021	DA-MIDL	AD, NC, pMCI, sMCI	ADNI, and AIBL	Accuracy: 92.4%, Sensitivity: 91%, Specificity: 93.80%, AUC: 96.50%, Specificity: 93.80%
[30], 2021	VGG16 and VGG19	MD, VMDMOD, ND	Alzheimer’s Dataset (4 class of Images)	Accuracy: 77.66% AUC: 81.55%, Precision: 58.48%, Recall: 36.67%, F1-score: 45.05% on VGG19
[54], 2021	VGG19 and DenseNet169	MD, VMDMOD, ND	Alzheimer’s Dataset (4 class of Images)	Accuracy: 82.6%, AUC 86.7%, on VGG19
[55], 2022	ADDTLA (CNN)	MD, MOD, ND, VMD	Alzheimer’s Dataset (4 class of Images)	Accuracy: 96%, Sensitivity: 96.57%, Specificity 98.29%
[56], 2023	CADx	(MCI), (CN)	ADNI	Accuracy: 92%, F1-score: 90%
**The Proposed Method**		MD, VMD, MOD, ND, and SD	AD5C	Accuracy: 98.24%, Precision: 97.8%. Recall 97.6%, F1-score: 98.2%

### Comparison with state-of-the-art techniques

In order to assess the efficacy of the proposed model, we applied TL to the DenseNet169 [54], Xception and InceptionV3 [40], VGG16 and VGG19 [30] models using the AD5C dataset. For this reason, all of the experiments utilized the same conditions and data enhancement strategies. In “Table” 8, we can see how precise contemporary DL methods have become. As shown in Table 8, the DensNet169 model obtained 92.85% accuracy; the Xception model obtained 96.92% accuracy; the inceptionV3 model carried off 93.06% accuracy; the VGG16 model obtained 77.66% accuracy; the VGG19 model acquired 94% accuracy. The proposed DensNet121 model acquired 98.24% accuracy on the AD5C dataset. We found that the proposed DenseNet121 model had the highest accuracy (98.24%) and that VGG16 had the lowest (77.66%) on the test set of the AD5C dataset, as compared to all the tested state-of-the-art models.

**Table 8:** Comparison with state-of-the-art methods.

Ref.	Methodology	Alzheimer’s Classes	Dataset	Accuracy
[54]	DenseNet169	MD, VMD, MOD, ND, and SD	AD5C	92.85%
[40]	Xception, InceptionV3	MD, VMD, MOD, ND, and SD	AD5C	Xception: 96.92% InceptionV3: 93.06%
[30]	VGG16, VGG19	MD, VMD, MOD, ND, and SD	AD5C	VGG16: 77.66%, VGG19: 94%
**The Proposed Method**		MD, VMD, MOD, ND, and SD	AD5C	98.24%

## Limitations of the study

Many researchers have found that utilizing deep learning algorithms to classify AD is effective. There are, however, some restrictions on this method. Important restrictions include the following: DL models need massive volumes of high-quality data to train properly. It cannot be easy to gather a comprehensive and accurately categorized dataset on AD. Getting massive longitudinal datasets, including medical images and DNA samples, is difficult. Due to data paucity, trained models may suffer from overfitting or lack of generalizability. They cannot be interpreted. Thus, figuring out what components or characteristics underlie AD classification is hard. In medicine, interpretability is essential for understanding illness causes and making informed treatment decisions. While DL models tend to excel in their training set, they may fail in novel or realistic settings. Variations in input data, such as imaging techniques, quality, or demography, may affect accuracy. Assessing deep learning models ’ robustness and generalizability is extremely important when using deep learning models in a clinical setting. Class imbalance is a common problem in AD datasets when the distribution of samples across categories is severely skewed. Because of this potential for bias, minority classes may be under-detected by deep learning algorithms. In order to achieve fair and accurate classification, it is necessary to deal with class imbalance using methods such as data augmentation or sample weighting.

## Conclusion and future work

AD is a neurological brain condition that causes memory decline over time. AD has no healing and cannot be reverted; thus, timely recognition is crucial. Different AD diagnosis methods are applied this way, but the MRI is the most useable neuroimaging technique to detect AD. The traditional methods are time-consuming and costly. Therefore, it can be overcome by using an affordable and automatic system for AD diagnosis. This research uses a TL method based on DenseNet-201 for MD, VMD, ND, MOD, and SD diagnosis. We can say that DenseNet-201 has demonstrated superior performance in AD classification with lesser computation cost. We believe using DenseNet-201 for AD classification offers a significant advantage over other methods, providing higher accuracy, less complexity, and resource utilization. It is observed that the multi-class DL approach performed significantly compared to binary classification techniques to accurately identify the different stages of AD. The major advantage of using multi-class DL techniques is the ability of the model to classify samples into multiple disease stages, such as MD, VMD, MOD, ND, and SD of AD. It helps the practitioners to identify the disease stage precisely, which helps to take the measures for appropriate treatment options.

Moreover, tang et al. compared the performance of binary and multi-class DL approaches for AD classification. The study’s findings show that the multi-class approach achieved higher classification accuracy than the binary approach, which indicates the superiority of the multi-class approach for AD classification. We can say that multi-class DL techniques have significant advantages over binary classification approaches in precisely and accurately identifying the stages of AD, leading to better treatment options and patient after-effects. The proposed technique was employed in an AD5C dataset of MRI images to determine different Alzheimer’s stages. The proposed method delivered excellent classification accuracy compared to other existing techniques. This model works efficiently and has an accuracy of 98.24%. Further research will emphasize different modalities for AD prediction and the segmentation of MRI images on a comparatively large dataset. Moreover, we can use a 3D dataset for efficiently Alzheimer’s detection and diagnosis.
