# Correction: Classification of Alzheimer disease using DenseNet-201 based on deep transfer learning technique

**DOI:** 10.1371/journal.pone.0321795

**Published:** 2025-04-02

**Authors:** Zsqa-ur-Rehman, Mohd Khalid Awang, Javed Rashid, Ghulam Ali, Muhammad Hamid, Samy F. Mahmoud, Dalia I. Saleh, Hafiz Ishfaq Ahmad

There is an error in the Acknowledgments section. The correct Acknowledgments section is as follows: The authors extend their appreciation to Taif University, Saudi Arabia, for supporting this work through project number (TU-DSPP-2024-53).
